# Cardiac magnetic resonance as a risk re-stratification tool in apical hypertrophic cardiomyopathy

**DOI:** 10.47487/apcyccv.v4i2.289

**Published:** 2023-06-30

**Authors:** Ana María Larriva, Sandra Rosales Uvera, Zuilma Vásquez, Beatriz Fernández, Diego Xavier Chango Azanza

**Affiliations:** 1 Servicio de Cardiología, Clínica Santa Ana, Cuenca, Ecuador. Servicio de Cardiología Clínica Santa Ana Cuenca Ecuador; 2 Servicio de Imagen Cardiovascular, Instituto Nacional de Ciencias Médicas y Nutrición «Salvador Zubirán», Cuidad de México, Mexico. Servicio de Imagen Cardiovascular Instituto Nacional de Ciencias Médicas y Nutrición «Salvador Zubirán Cuidad de México Mexico

**Keywords:** Apical Hypertrophic Cardiomyopathy, Cardiac Aneurysm, Echocardiography, Cardiac Magnetic Resonance, Miocardiopatía Hipertrófica Apical, Aneurisma Cardiaco, Ecocardiografía, Resonancia Magnética

## Abstract

Apical hypertrophic cardiomyopathy (ApHCM) can result in the formation of a left ventricular apical aneurysm and progressive myocardial fibrosis, which is associated with a worse prognosis. We present the case of a 76-year-old man previously diagnosed with ApHCM seven years ago, who has been under clinical follow-up. Serial cardiac magnetic resonance (CMR) imaging was performed in 2013 and 2020 due to suspected apical aneurysm formation based on echocardiographic evaluation. The 2020 CMR imaging revealed an increase in myocardial fibrosis observed through late-gadolinium enhancement images and, for the first time, a small apical aneurysm that was not clearly visualized on two-dimensional echocardiography. The time course leading to the development of an ApHCM aneurysm is not well-defined and may impact the clinical course.

## RESUMEN

La miocardiopatía hipertrófica apical (MCHap) puede provocar la formación de un aneurisma apical del ventrículo izquierdo (LV) y una fibrosis miocárdica progresiva que se relaciona con un peor pronóstico. Se presenta el relato de un paciente de 76 años con diagnóstico previo de MCHap hace siete años en seguimiento clínico. Se realizó una resonancia magnética cardíaca (RMC) seriada en 2013 y 2020, ante la sospecha de formación de aneurisma apical mediante ecocardiografía. Las imágenes RMC del 2020 demostraron un aumento de la fibrosis miocárdica mediante imágenes de realce tardío con gadolinio y, por primera vez, un pequeño aneurisma apical que no fue definido en forma precisa en la ecocardiografía bidimensional. El tiempo de progresión hasta el desarrollo del aneurisma en la MCHap no está claramente definido y puede relacionarse con cambios en el curso clínico.

## Introduction

Hypertrophic cardiomyopathy (HCM) is a disease that results in primary left ventricular (LV) hypertrophy, presenting with various morphological subtypes. The genetic etiology of this condition involves mutations in genetic sarcomeric proteins ^1^. The apical HCM (ApHCM) phenotype is less common than the classic presentation and is more sporadic in nature. The presence of sarcomeric mutations is less frequent, and there is a different prevalence of atrial fibrillation (AF) and sudden cardiac death (SCD). Current guidelines lack specific recommendations concerning the diagnosis, indications for family screening, and risk stratification of ApHCM ^2^. An apical aneurysm is characterized by a discrete, thin-walled, dyskinetic/akinetic segment in the distal parts of the LV that exhibits wide communication with the large cavity during diastole ^3^. The prevalence of apical aneurysms is 13% in HCM and 15% in patients with ApHCM, and they can be associated with adverse cardiac events ^3^^,^^4^.

## Case report

**Figure 1:**
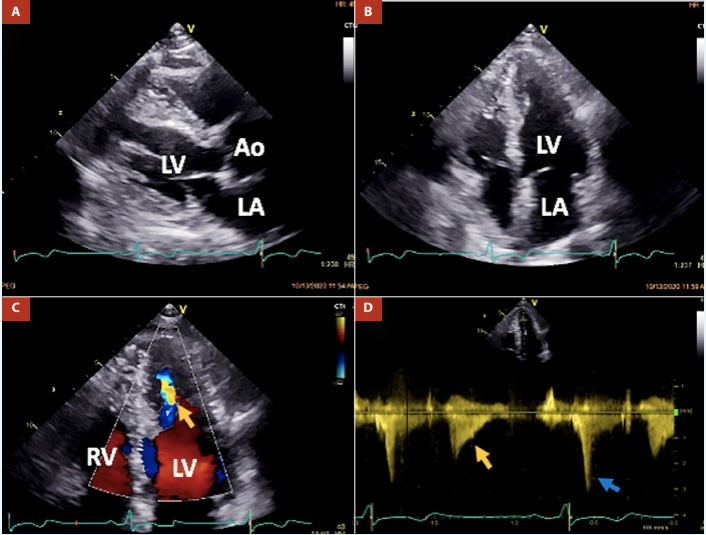
Main findings at transthoracic echocardiography in the patient. Panels A and B demonstrate hypertrophy of both the ventricular septum and lateral wall at mid-ventricular and apical levels in parasternal long-axis and apical four-chamber views, respectively. Panels C and D depict a mid-ventricular paradoxical jet flow from the apical to the basal chamber in diastole (indicated by orange arrows) and dynamic obstruction caused by head-on septum to lateral wall motion and high intra-apical end-systolic pressure (indicated by the blue arrow).

**Figure 2:**
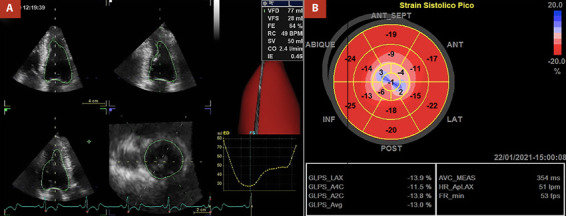
Determination of left ventricular (LV) systolic function parameters. Panel A shows a three-dimensional echocardiography image with normal LV volumes and ejection fraction. Panel B displays an LV global longitudinal strain map with altered myocardial deformation in the hypertrophic LV segments

Herein, we present the case of a 76-year-old male who has been undergoing clinical and periodic imaging follow-ups since his diagnosis of ApHCM in 2013. He remained asymptomatic for dyspnea, chest pain, palpitations, and arrhythmic episodes. In a two-dimensional echocardiography examination conducted in 2020, normal LV basal thickness was observed, with hypertrophy involving the septum and lateral walls at mid-ventricular and apical levels, resulting in mid-cavity obliteration. Paradoxical dynamic obstruction with a systolic jet flow from the basal LV chamber to the apex (and vice versa in diastole) was detected (Figure 1). Three-dimensional echocardiography indicated normal LV volumes and a normal ejection fraction of 64%. However, global longitudinal strain (GLS) was abnormally estimated at -13%, indicating altered deformation in the apical segments within the context of ApHCM (Figure 2).

AO: aortic valve, LA: left atrium, LV: left ventricle, RV: right ventricle.

**Figure 3:**
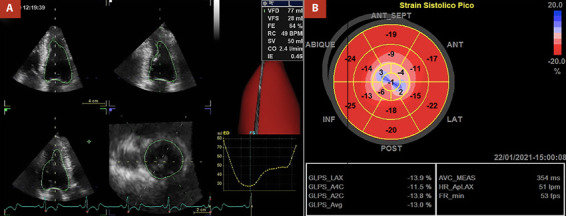
Findings at the 2020 cardiac magnetic resonance (CMR) examination in the patient. Panel A is a CINE image (end-diastolic frame) demonstrating hypertrophy of both the ventricular septum (maximal wall thickness 18.6 mm) and lateral wall at mid-ventricular and apical levels in the five-chamber view. Panel B is a CINE image (end-systolic frame) illustrating mid-ventricular dynamic obstruction caused by head-on septum to lateral wall motion (indicated by red asterisks) and the presence of a small apical aneurysm (indicated by an orange arrow).

These findings raised suspicion of apical aneurysm formation, although it could not be definitively confirmed through two-dimensional echocardiography. Therefore, a further assessment was conducted using CMR imaging, which revealed the presence of a small apical aneurysm that was not observed in the previous examination in 2013 (Figure 3).

AO: aortic valve, LA: left atrium, RA: right atrium, LV: left ventricle, and RV: right ventricle.

**Figure 4:**
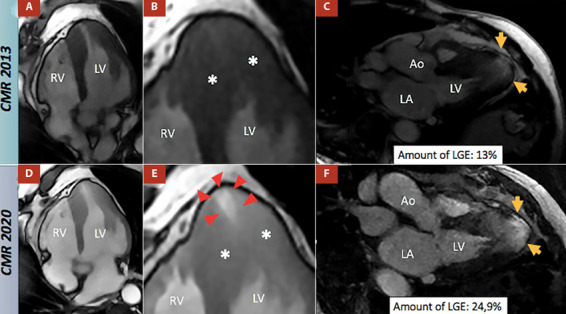
Comparative images from CMR 2013 (Panels A, B, and C) to 2020 (Panels D, E, and F) in the patient. Panels A and B display the 2013 CINE images at end-diastole and end-systole, respectively. In the end-systolic frame (Panel B), mid-ventricular dynamic obstruction caused by head-on septum to lateral wall motion is observed (indicated by black asterisks), and no apical aneurysm is present. Panel C shows a late-gadolinium enhancement image with the presence of fibrosis in the hypertrophic walls, estimated to be 13% of LV mass (indicated by a yellow arrow). Panels D and E depict the 2020 CINE images at end-diastole and end-systole. In the end-systolic frame (Panel E), mid-ventricular dynamic obstruction caused by head-on septum to lateral wall motion is observed (indicated by black asterisks), along with the formation of a small apical aneurysm (indicated by red arrows). Panel F demonstrates progressive fibrosis and an increase in the amount of fibrosis to 24.9% of LV mass (indicated by a yellow arrow).

Comparing the CMR findings from 2013 and 2020, a progressive extension of myocardial fibrosis was observed, with a higher amount of late-gadolinium enhancement estimated to have increased from 13% to 24.9%. Maximal wall thickness was estimated at the septal-apical segment (18.4 mm), and no significant changes were observed in LV mass (from 123 g to 130 g) and LVEF (from 64% to 60%) (Figure 4).

CRM: cardiac magnetic resonance, LGE: late gadolinium enhancement, AO: aortic valve, LA: left atrium, LV: left ventricle, and RV: right ventricle.

In our patient, new apical aneurysm detection allowed oral permanent anticoagulants indication without any thromboembolic or cardiovascular events. The patient remained asymptomatic under clinical, one-year follow-up.

## Discussion

Despite being less frequent compared to other types of HCM, the ApHCM phenotype is not as rare as initially believed, accounting for up to 25% of HCM in Asian populations and 1% to 10% in non-Asians ^5^. The clinical presentation of ApHCM is characterized by the absence of apical tapering and classical changes in the electrocardiogram, such as precordial T-wave inversion. Therefore, the diagnosis was made based on imaging evidence of LV hypertrophy predominantly involving the apex, which can be defined as a wall thickness ≥15 mm or a ratio of maximal apical to posterior wall thickness ≥1.5 as determined by echocardiography or CMR imaging.

In contrast to classic HCM, ApHCM does not typically exhibit left ventricular outflow tract obstruction resulting from systolic anterior motion of the mitral valve and is not associated with concomitant mitral regurgitation. However, midventricular obstruction and cavity obliteration may or may not be present, accompanied by the formation of an apical aneurysm. Mid-ventricular obstruction arises from hypertrophy of the mid-apical lateral and septal regions ^6^^,^^7^. In cases where hypertrophy is significant, cavity obliteration and mid-ventricular obstruction persist during diastole, resulting in a paradoxical mid-cavity diastolic flow jet. This phenomenon is consistent with the presence of an apical aneurysm ^3^. The paradoxical flow, which occurs from the base to the apex during diastole, initiates in isovolumetric relaxation and persists for nearly 60% of diastole. This flow pattern is associated with a reduction in the size of the apical cavity during diastole while the base-to-apex mitral inflow demonstrates a marked increase in the size of the basal portion of the LV. The apical cavity fills during late diastole, after the cessation of diastolic flow from apex to base, and during isovolumetric systole. During systolic ejection, the flow from apex-to-the-base is either abruptly halted by midventricular obstruction or attenuated during the latter half of systole ^8^.

The presence of apical aneurysms and obstruction can be attributed to regional myocardial scarring, which arises due to repeated exposure of the apical region to increased LV wall stress, high systolic pressures, elevated oxygen consumption, altered coronary perfusion, and the ischemia. Consequently, dyskinetic/akinetic aneurysms pose a risk of apical thrombus formation and thromboembolic events ^4^. Additionally, apical aneurysms have been associated with increased hypertrophic severity, SCD, monomorphic ventricular tachycardia ^9^, LV systolic dysfunction, and heart failure.

The use of CMR imaging allows for accurate phenotypic characterization of HCM, enabling clarification of inconclusive cases from echocardiography and the exclusion of alternative diagnoses as outlined in the 2020 guidelines. CMR imaging is a valuable technique for evaluating maximal wall thickness, LV ejection fraction, the presence of LV apical aneurysms, and the extent of myocardial fibrosis for the purpose of SCD risk stratification ^10^.

The size of the aneurysm does not consistently correlate with clinical outcomes. Even in small aneurysms, approximately 20% of patients experience thromboembolic events and apical clot formation. Therefore, anticoagulation should be considered in all patients with aneurysms, regardless of the size. No embolic events or apical thrombus formation have been reported during the follow-up period when prophylactic anticoagulation was administered to these patients ^11^. However, the risk of SCD remains in approximately 70% of patients with medium-to-large aneurysms ^12^.

Despite the absence of definitive data regarding the development of aneurysm apical formation in ApHCM current guidelines recommend serial CMR evaluation for re-stratification of these patients. Non-specific trials were developed by repeating systemically CMR imaging to elucidate the timeline of apical aneurysm formation. In a recent pilot serial CMR study of 76 patients with ApHCM, aneurysm formation was related to four stages for its development, starting with systolic apical cavity obliteration, then broadening of the apical slit in systole, further developing into an apical outpouching, and finally forming an apical aneurysm. Nevertheless, further investigations are required to clarify time progression and established specific CMR follow-up recommendations.

In conclusion, apical aneurysm detection in ApHCM is a valuable strategy for identifying patients at higher risk of cardiovascular events. Echocardiography is the primary modality for assessment and monitoring, while CMR imaging plays a confirmatory role in inconclusive cases. Regular imaging follow-up is recommended to detect aneurysm formation and assess the progression of myocardial fibrosis, enabling re-evaluation of the risk of SCD. In this case, CMR imaging played a crucial role in re-stratification.
